# Upregulation of MicroRNA-125b Leads to the Resistance to Inflammatory Injury in Endothelial Progenitor Cells

**DOI:** 10.1155/2020/6210847

**Published:** 2020-09-14

**Authors:** Ke Yang, Xing Liu, Wanwen Lin, Yuanyuan Zhang, Chaoquan Peng

**Affiliations:** Department of Cardiology, The Third Affiliated Hospital, Sun Yat-sen University, Tian-he Road, Guangzhou 510630, China

## Abstract

**Objectives:**

MicroRNA-125b (miR-125b) has been recognized as one of the key regulators of the inflammatory responses in cardiovascular diseases recently. This study sought to dissect the role of miR-125b in modulating the function of endothelial progenitor cells (EPCs) in the inflammatory environment of ischemic hearts.

**Methods:**

EPCs were cultured and transfected with miR-125b mimic and negative control mimic. Cell migration and adhesion assays were performed after tumor necrosis factor-*α* (TNF-*α*) treatment to determine EPC function. Cell apoptosis was analyzed by flow cytometry. The activation of the NF-*κ*B pathway was measured by western blotting. EPC-mediated neovascularization in vivo was studied by using a myocardial infarction model.

**Results:**

miR-125b-overexpressed EPCs displayed improved cell migration, adhesion abilities, and reduced cell apoptosis compared with those of the NC group after TNF-*α* treatment. miR-125b overexpression in EPCs ameliorated TNF-*α*-induced activation of the NF-*κ*B pathway. Mice transplanted with miR-125b-overexpressed EPCs showed improved cardiac function recovery and capillary vessel density than the ones transplanted with NC EPCs.

**Conclusions:**

miR-125b protects EPCs against TNF-*α*-induced inflammation and cell apoptosis by attenuating the activation of NF-*κ*B pathway and consequently improves the cardiac function recovery and EPC-mediated neovascularization in the ischemic hearts.

## 1. Introduction

The role of EPCs in vascular and tissue repair in ischemic conditions, such as coronary or peripheral vascular diseases, has been well recognized [1]. Circulating EPCs are recruited into the ischemic sites, and they enhance repair through paracrine effects or by incorporating into newly formed vessels after ischemic injury [2–4]. Of note, the functional activities of EPCs are impaired in patients with coronary artery disease (CAD). Compelling evidence suggests that the number and function of EPCs inversely correlate with risk factors for CAD, such as hypertension, diabetes, dyslipidemia, smoking, and age [5–7]. Furthermore, the hostile inflammatory environment in the ischemic sites can induce the apoptosis of EPCs and, consequently, impede the EPC-mediated repair [8, 9]. Hence, improving the function and survival of EPCs in the ischemic sites is critical for EPC-mediated repair.

MicroRNAs (miRNAs) are known as a class of noncoding RNAs that modulate the gene expression at a posttranscriptional level. miR-125, known as one of the major regulators in the development of hematological malignancies, is a family of highly conserved miRNAs throughout diverse species [10]. Emerging evidence suggests that miR-125 is involved in the regulation of the innate immune and inflammatory responses [11, 12]. More importantly, the role of miR-125b in cardiovascular diseases has been drawing increasing attention recently. Dr. Wang et al. reported that the target of miR-125b in the mouse heart is TNF receptor-associated factor 6 (TRAF6), an adaptor molecule in the NF-kB pathway. Overexpression of miR-125b in the mouse heart protects the myocardium from ischemia/reperfusion injury by suppressing the TRAF6-mediated NF-kB activation [13]. However, the role of miR-125b in the regulation of EPCs is still unclear, and further studies are required to study its role.

In the present study, we focused on the role of miRNA-125b in regulating the inflammatory response and function of EPCs in the ischemic hearts. Our results confirmed that upregulation of miRNA-125b ameliorated TNF-*α*-induced functional defects in EPCs in vitro and enhanced EPC-mediated neovascularization in the ischemic hearts. miR-125b-mediated inhibition of TNF-*α*/NF-*κ*B pathway activation is involved in the beneficial effects we observed.

## 2. Methods and Materials

### 2.1. Cell Culture and miRNA Transfection

Bone marrow mononuclear cells were isolated by density gradient centrifugation from the mouse bone marrow and cultured in endothelial cell basal medium-2 (EBM-2) supplemented with endothelial growth medium SingleQuots as indicated by the manufacturer (Clonetics, San Diego). After 4 days of culture, nonadherent cells were removed by washing the plates with phosphate-buffered solution (PBS), and a new medium was applied. EPCs were transfected for 24 h on day 6 with 50 nmol/L microRNA mimics for miR-125b (MC10148, Ambion) or miR-negative control (AM17010, Ambion), using the Lipofectamine RNAiMAX reagent (Invitrogen) according to the manufacturer's protocol [13]. Cells were treated with or without 10 ng/mL tumor necrosis factor-*α* (TNF-*α*, Peprotech) for 1 h and then used for the following experiments at day 7. Cultured EPCs were identified by the flow cytometry analysis. Based on the isolation and cultivation protocol, the adherent mononuclear cells were identified as EPCs similar to the previous studies.

### 2.2. EPC Adhesion to Endothelial Cells In Vitro

2 × 10^5^ human umbilical vein endothelial cells (HUVECs) were seeded in each well of a four-well plate 48 h before the assay to prepare a monolayer of ECs. Then, 1 × 10^5^ EPCs labelled with CM-DiI (CellTrackerTM CM-DiI, Invitrogen) were added to each well and incubated for 3 h at 37°C. Nonattached cells were gently washed and removed with PBS, and adherent EPCs were fixed with 4% paraformaldehyde and counted by independent investigators blinded to treatment randomly.

### 2.3. EPC Migration In Vitro

A total of 2 × 10^4^ EPCs were harvested and resuspended in 250 *μ*L EBM-2 after TNF-*α* treatment and pipetted into the upper chamber of a modified Boyden chamber (Costar Transwell assay, 8 *μ*m pore size, Corning, NY), which was placed in a 24-well culture plate containing 500 *μ*L EBM-2 medium supplemented with 100 ng/mL SDF-1. Transmigrated cells were counted after 24 h incubation at 37°C by independent investigators blinded to the treatment randomly.

### 2.4. EPC Apoptosis Assay

EPCs were treated with 10 ng/mL tumor necrosis factor-*α* (TNF-*α*, Peprotech) for 1 h, and cell apoptosis was detected by AnnexinV-staining (Roche, Penzberg, Germany). Briefly, EPCs were cultured with TNF-*α* (10 ng/mL) for 1 h. Then, EPCs were collected and washed for three times. Annexin V-FITC and propidium iodide (PI) were added to the washed cells (1 × 10^6^ cells/mL in FACS buffer) for 15 min at room temperature in the dark. FACS buffer was added, and cells were analyzed immediately by flow cytometry analysis.

### 2.5. Quantitative Real-Time Reverse Transcription Polymerase Chain Reaction

miR-125b-5p were quantified using specific Taqman assays for miR (Applied Biosystems, USA). Specific primers for miR-125b-5p were obtained from Applied Biosystems. miR-125b levels were normalized to the U6 small nucleolar RNA. Primer sequences for gene encoding for TNF-*α*, IL-1*β*, IL-6, and *β*-actin were reported in Table 1. The results were normalized to the mRNA levels of *β*-actin.

### 2.6. Western Blotting

Proteins were extracted with cell lysis buffer (Cell Signaling Technology) and analyzed with by western blotting by using p-NF*κ*B p65 antibody (Ser 276) (1 : 1000, Santa Cruz, sc-101749), NF-*κ*B p65 (1 : 1000, Cell Signaling, 8242T) and rabbit anti-*β*-actin antibodies (1 : 3000; Cell Signaling Technology). The intensities of protein bands were quantified densitometrically by using the NIH IMAGE J software.

### 2.7. Surgical Induction of Myocardial Infarction (MI) and EPC Transplantation

The mice were anesthetized by 5.0% isoflurane, and anaesthesia was maintained by inhalation of 1.5% to 2% isoflurane driven by oxygen flow using a rodent ventilator. The hearts were exposed, and the left anterior descending (LAD) coronary artery was ligated with an 8-0 silk ligature. 2 × 10^5^ EPCs suspended in 20 *μ*l PBS were injected at 5 different sites at the infarct border zone using an 20 *μ*l Hamilton syringe with a 30-gauge needle. 6–8 mice were used for LAD ligation in each group. The cardiac function was evaluated by echocardiography. The study protocol was approved by the Ethics Committee of Sun Yat-sen University.

### 2.8. Histological Assessments

Cardiac tissues were fixed in 4% paraformaldehyde for 4 hours and then snapfrozen. Serial cryosectioning was performed starting at 1 mm below the LAD ligation moving toward the apex. To evaluate PC endothelial differentiation and capillary density, immunohistochemical staining was performed using fluorescent anti-CD31 (Santa Cruz) antibodies. All surgical procedures and pathohistological analyses were performed by investigators blinded to treatment assignments.

### 2.9. Statistical Analyses

All values are reported as mean ± SEM. Two-tailed Student's *t* test was used to compare 2 means. One-way or 2-way ANOVA with a Bonferroni correction was used to compare multiple (>2) means with 1 or 2 independent variables, respectively. *p* < 0.05 was considered significant.

## 3. Results

### 3.1. Overexpression of miRNA-125b in EPCs Ameliorates TNF-*α* Induced Functional Defects in EPCs

**Figure 1:**
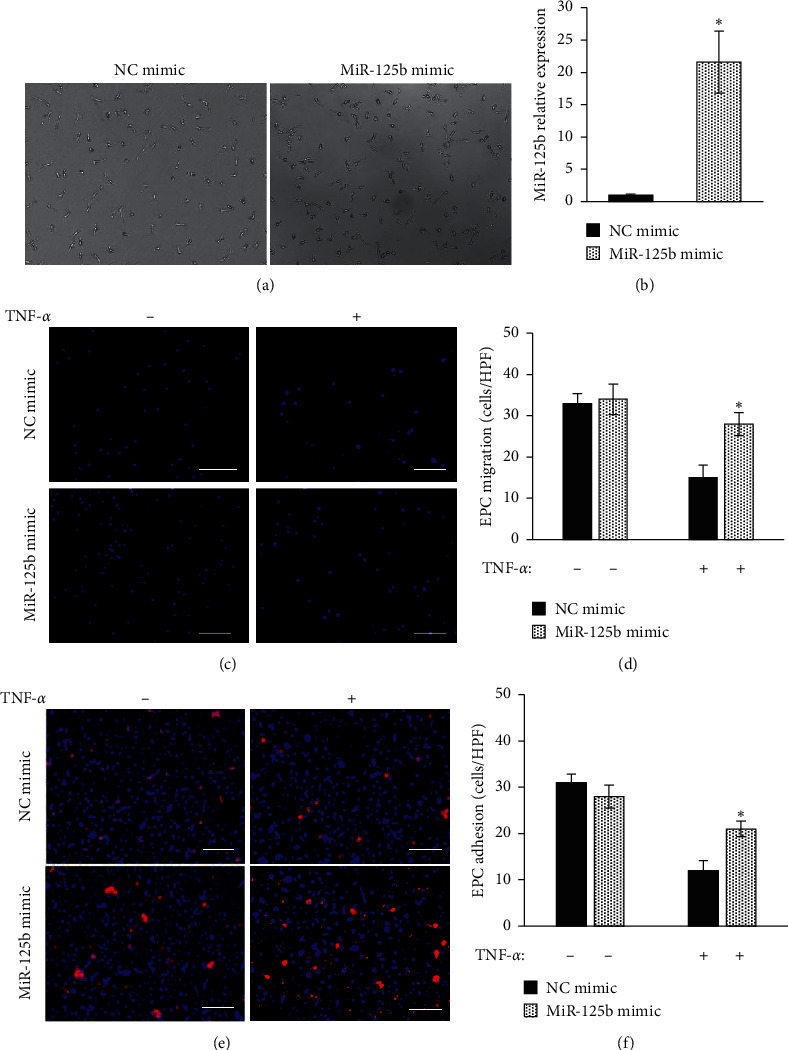
miR-125b overexpression in EPCs preserved its migration and adhesion function after TNF-*α* treatment. EPCs were transfected with miR-125b mimic and negative control mimic for 24 h. (a) The level of miR125b measured by qRT-PCR (b). EPC migration and adhesion measured after transfection. Representative (c) and quantification (d) of the migratory activity of EPCs. Representative (e) and quantification (f) of DiI-labeled EPC adhesion to HUVECs with TNF-*α* activation (scale bar = 100 *μ*m, ^*∗*^*p* < 0.05 vs. NC mimic with TNF-*α* treatment, *n* = 5).

The level of miR-125b in the EPC transfected with miR125b mimic is about 21-folds increase compared with the negative control (NC) group (Figures 1(a) and 1(b)). Transfected EPCs were treated with or without 10 ng/mL TNF-*α* for 1 h and then tested for cell migration and adhesion in vitro. The NC group showed significant reduced cell migration (Figures 1(c) and 1(d)) and adhesion (Figures 1(e) and 1(f)) capacity after TNF-*α* treatment compared with the one without TNF-*α* treatment, while TNF-*α* treatment slightly reduced the cell migration and adhesion function in the miR-125b-overexpressed group in comparison with cells without TNF-*α* treatment. After TNF-*α* treatment, miR-125b-overexpressed EPCs showed significantly better preserved migration and adhesion function than those of the NC group.

### 3.2. Overexpression of miRNA-125b in EPCs Attenuates TNF-*α* Induced Expression of Proinflammatory Factors and Cell Apoptosis

**Figure 2:**
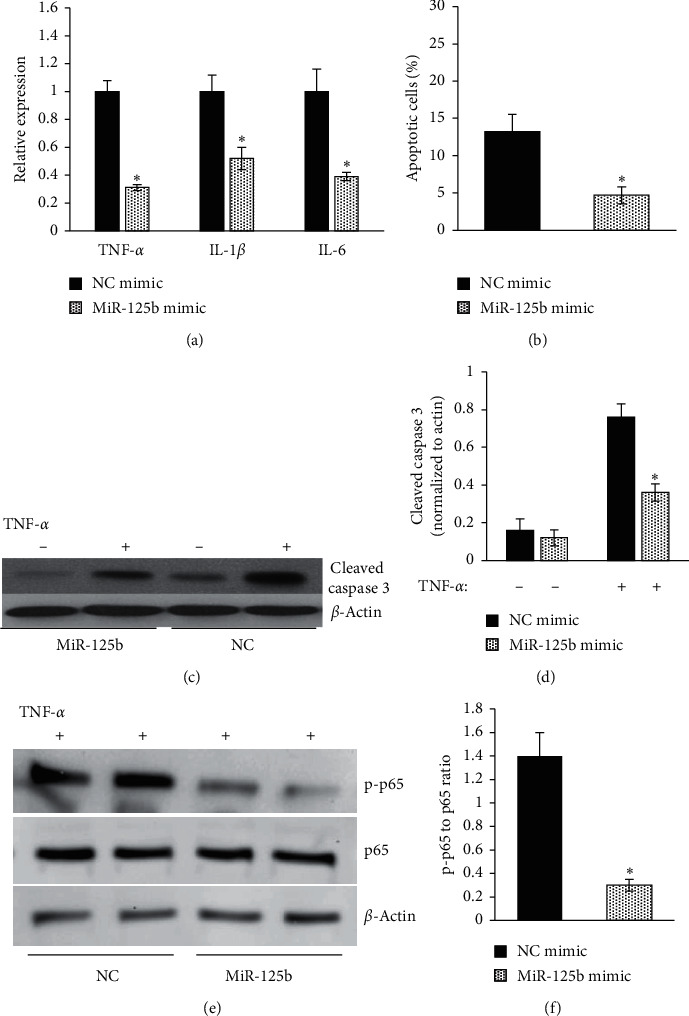
Upregulation of miR-125b in EPCs attenuated the expression of proinflammatory factors and ameliorated the TNF-*α*-induced cell apoptosis. EPCs were treated with TNF-*α* (10 ng/mL) for 1 h after transfection. The mRNA levels of proinflammatory factors (TNF-*α*, IL-1*β*, and IL-6) were measured by qRT-PCR (a). Cell apoptosis was determined by flow cytometry using annexin V staining (b). The activation of caspase3 was analyzed by western blotting. Representative (c) and quantification (d) of caspase3 level (normalized to *β*-actin). The activation of NF-*κ*B was determined by the level of p-p65 in EPCs using western blotting. Representative (e) and quantification (f) of p-p65 level (normalized to p65) (^*∗*^*p* < 0.05 vs. NC mimic with TNF-*α* treatment, *n* = 5).

The mRNA level of the proinflammatory cytokines (TNF-*α*, IL-1*β*, and IL-6) in EPCs was measured by qRT-PCR after 1 h TNF-*α* treatment. We found that miR-125b-overexpressed EPCs showed significantly lower TNF-*α*, IL-1*β*, and IL-6 mRNA expression levels than those of the NC group (Figure 2(a)). Moreover, the percentage of apoptotic cells (Annexin V positive cells) is significantly lower in the miR-125b-overexpressed group compared with that of the NC group after TNF-*α* treatment (Figure 2(b)). In parallel with these flow cytometry results, our western blotting results showed markedly lower cleaved caspase 3 level in the overexpression group than in the NC group after TNF-*α* treatment (Figures 2(c) and 2(d)) which indicated reduced cell apoptosis.

### 3.3. Effects of miRNA-125b on EPC-Mediated Neovascularization in the Ischemic Hearts

**Figure 3:**
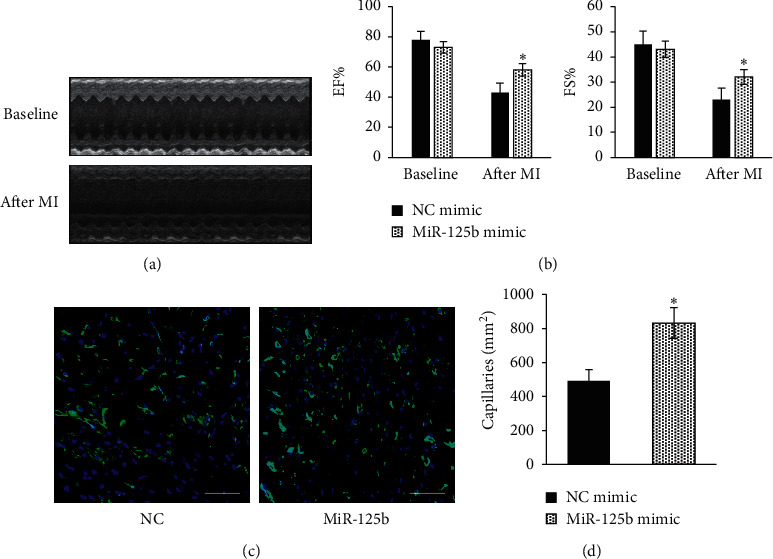
miR-125b overexpression enhanced the EPC-mediated neovascularization and cardiac function recovery in the ischemic hearts. The cardiac function was assessed by echocardiography at baseline and after MI (28 days). Representative (a) and quantification (b) of echocardiography analyses. EPC-mediated neovascularization in the ischemic hearts analyzed by CD31 staining (GFP) (c) and capillary density quantified (d) (scale bar = 100 *μ*m, ^*∗*^*p* < 0.05 vs. NC mimic, *n* = 5).

To investigate the effect of miR-125b on the regulation of TNF-*α* induced NF-*κ*B pathway activation, western blotting was performed to analyze the level of p65 phosphorylation (p–p65). As shown in Figures 2(e) and 2(f), the level of p–p65 in miR-125b-overexpressed EPCs was markedly lower than of the NC group after TNF-*α* treatment. To further investigate the role of miRNA-125b in EPC-mediated neovascularization, miR-125b-overexpressed and NC control EPCs were transplanted by intramyocardial injections into the mice immediately after surgical-induction of MI, the cardiac function was evaluated by echocardiography, and the capillary density in the infarct border zone was assessed by immunohistology staining of CD31. The cardiac function of the mice transplanted with miR-125b-overexpressed EPCs was improved compared with that of the ones transplanted with NC control EPCs 28 days after MI (Figures 3(a) and 2(b)). The capillary density in the infarct border zone of the mice transplanted with miR-125b-overexpressed EPCs was about 2-folds higher than the ones transplanted with NC control EPCs (Figures 3(c) and 2(d)). These data suggest miR-125b overexpression in EPC-enhanced EPC-mediated neovascularization and cardiac function recovery in the ischemic hearts.

## 4. Discussion

In this study, we have identified a novel role of miR-125b in the regulation of EPC functions. Upregulation of miR-125b in EPCs ameliorates the inflammatory and apoptotic responses of EPCs in the ischemic heart by inhibiting the activation of the TNF-*α*/NF-*κ*B pathway. Using the myocardial infarction model, we demonstrated that miR-125b overexpression enhanced the EPC-mediated neovascularization and cardiac function recovery in the ischemic hearts. To the best of our knowledge, this is the first study to address the role of miR-125b in modulating EPC-mediated neovascularization in the ischemic hearts.

Although EPC-related cell therapy has been studied extensively, the majority of the cell-therapy trials achieve only modest efficacy [14–16]. The low survival rate of the transplanted EPCs in the ischemic hearts is one of the major obstacles to the success of this therapy [17, 18]. After the myocardial infarction, a large number of inflammatory cells are recruited to the ischemic heart where a hostile, pro-inflammatory environment is created. Compelling evidence showed that the EPC level can be significantly affected by systemic inflammation [19]. It has been reported that lipopolysaccharide-induced systemic inflammatory reaction led to a decrease in the number of circulating EPCs [20]. Of note, patients with long-term inflammatory disease, like active ulcerative colitis, had significantly lower levels of circulating EPCs [21]. In this study, our data indicate that TNF-*α* treatment in EPCs markedly impaired its migration and adhesion function. More importantly, EPCs treated with TNF-*α* showed increased cell apoptosis, which might partly explain the low survival rate of transplanted EPCs in the ischemic hearts. Collectively, others and our study suggest that enhancing the survival of EPCs in the proinflammatory environment in the ischemic hearts is crucial for achieving satisfactory outcome of cell therapy.

miR-125 family is well known as one of the major regulators in the development of hematological malignancies and autoimmune diseases [22–24]. Recently, accumulating studies demonstrate that miR-125b negatively regulates the activation of the NF-*κ*B pathway by targeting TRAF6 [25]. More important, evidence showed that miR-125b-mediated repression of the NF-*κ*B pathway exerts a protective effect on ischemic hearts [13]. However, the role of miR125b in the regulation of EPC function is still unclear. Our study for the first time demonstrated that the overexpression of miR-125b led to resistance to TNF-*α*-induced functional defects and cell apoptosis in EPCs and consequently enhanced the EPC-mediated neovascularization in the ischemic hearts. It is well recognized that the TNF-*α*/NF-*κ*B pathway is central to most of the inflammatory processes and exerts negative regulatory effect on vascular repair [26]. In consistency with the reports from other cell types [27], our data showed that the overexpression of miR-125b blunted the TNF-*α* induced proinflammatory responses in EPCs and restored the functions of EPCs and, more importantly, protected against TNF-*α* induced apoptosis. Furthermore, mice transplanted with miR-125b-overexpressed EPCs showed enhanced neovascularization compared with that of the one with NC control EPCs. Taking together, our study for the first time unveils the protective effect of miR-125b on EPCs.

Although our data have demonstrated that upregulation of miR-125b blunted the TNF-*α*-induced NF-*κ*B pathway activation in EPCs, the detailed mechanisms underlying miR-125b-mediated negative regulation of NF-*κ*B pathway in EPCs has not been revealed in this study. Reports from others have characterized TRAF6 as the target of miR-125b in the NF-*κ*B pathway activation [13, 25, 27]. However, whether miR-125b exerts its effect on TNF-*α*/NF-*κ*B pathway by targeting TRAF6 or other molecules in EPCs still requires further investigations to confirm.

In conclusion, our study suggests that miR-125b-mediated inhibition of the TNF-*α*/NF-*κ*B pathway is crucial for the protection of EPCs in the inflammatory environment and may be a novel therapeutic target for enhancing the effectiveness of cell therapy for ischemic heart disease.

## Figures and Tables

**Table 1:** Primer sequences.

Gene	Forward primer	Reverse primer
TNF-alpha	AGGGATGAGAAGTTCCCAAATG	AGGGATGAGAAGTTCCCAAATG
IL-1*β*	GCAACTGTTCCTGAACTCAACT	ATCTTTTGGGGTCCGTCAACT
IL-6	TCGGAGGCTTAATTACACATGTTC	TGCCATTGCACAACTCTTTTCT

## Data Availability

The data used to support the findings of this study are included within the article.
